# Predicting clinically significant prostate cancer following suspicious mpMRI: analyses from a high-volume center

**DOI:** 10.1007/s00345-024-04991-6

**Published:** 2024-05-03

**Authors:** Matthias Jahnen, Tanja Hausler, Valentin H. Meissner, Donna P. Ankerst, Michael W. Kattan, Andreas Sauter, Juergen E. Gschwend, Kathleen Herkommer

**Affiliations:** 1 Department of Urology, School of Medicine and Health, Technical University of Munich (TUM) Rechts der Isar University Hospital, Ismaningerstr. 22, 81675 Munich, Germany; 2 Department of Mathematics, School of Computation, Information, and Technology, Boltzmannstr. 3, 85748 Garching, Germany; 3 Department of Quantitative Health Sciences, Cleveland Clinic, Cleveland, OH 44195 USA; 4 Department of Diagnostic and Interventional Radiology, School of Medicine and Health, Technical University of Munich (TUM) Rechts der Isar University Hospital, Ismaningerstr. 22, 81675 Munich, Germany

**Keywords:** Prostate cancer, mpMRI, Prostate biopsy, Risk models

## Abstract

**Purpose:**

mpMRI is routinely used to stratify the risk of clinically significant prostate cancer (csPCa) in men with elevated PSA values before biopsy. This study aimed to calculate a multivariable risk model incorporating standard risk factors and mpMRI findings for predicting csPCa on subsequent prostate biopsy.

**Methods:**

Data from 677 patients undergoing mpMRI ultrasound fusion biopsy of the prostate at the TUM University Hospital tertiary urological center between 2019 and 2023 were analyzed. Patient age at biopsy (67 (median); 33–88 (range) (years)), PSA (7.2; 0.3–439 (ng/ml)), prostate volume (45; 10–300 (ml)), PSA density (0.15; 0.01–8.4), PI-RADS (V.2.0 protocol) score of index lesion (92.2% ≥3), prior negative biopsy (12.9%), suspicious digital rectal examination (31.2%), biopsy cores taken (12; 2–22), and pathological biopsy outcome were analyzed with multivariable logistic regression for independent associations with the detection of csPCa defined as ISUP ≥ 3 (*n* = 212 (35.2%)) and ISUP ≥ 2 (*n* = 459 (67.8%) performed on 603 patients with complete information.

**Results:**

Older age (OR: 1.64 for a 10-year increase; *p* < 0.001), higher PSA density (OR: 1.60 for a doubling; *p* < 0.001), higher PI-RADS score of the index lesion (OR: 2.35 for an increase of 1; *p* < 0.001), and a prior negative biopsy (OR: 0.43; *p* = 0.01) were associated with csPCa.

**Conclusion:**

mpMRI findings are the dominant predictor for csPCa on follow-up prostate biopsy. However, PSA density, age, and prior negative biopsy history are independent predictors. They must be considered when discussing the individual risk for csPCa following suspicious mpMRI and may help facilitate the further diagnostical approach.

**Supplementary Information:**

The online version contains supplementary material available at 10.1007/s00345-024-04991-6.

## Introduction

Prostate-specific antigen (PSA) based early detection for prostate cancer (PCa) has been shown to potentially reduce PCa-related death [1]. However, it is also associated with overdiagnosis of clinically non-significant PCa and unnecessarily performed invasive biopsies [1, 2]. The introduction of multi-parametric magnetic resonance imaging (mpMRI) of the prostate in men with elevated PSA value has substantially facilitated clinical decision-making regarding prostate biopsy and reduced the detection of clinically non-significant PCa defined as International Society of Urological Pathology classification (ISUP) grade  1 [3, 4]. mpMRI is routinely used to stratify the risk of clinically significant PCa (csPCa) before biopsy, using the Prostate Imaging Reporting and Data System (PI-RADS) [5]. In this regard, mpMRI can reliably exclude the presence of csPCa in the case of nonsuspicious lesions [3, 5, 6]. Further, in the case of suspicious lesions classified with a PI-RADS score ≥ 3 csPCa becomes likely, warranting further diagnostic consideration [3, 5]. Moreover, these suspicious mpMRI lesions may be used for MRI-targeted biopsy, leading to an improved detection rate of csPCa [7–9].

Nevertheless, the positive predictive value of a suspicious mpMRI is subject to considerable variability when comparing previous studies, making mpMRI-based csPCA risk prediction difficult [10]. Even after stratifying suspicious mpMRIs with regard to the PI-RADS classification, evident variability remains across different studies. The detection rate of csPCa in men with a PI-RADS 3, 4, and 5 index lesion has been reported to range between 7 and 27%, 39–78%, and 73–94%, respectively [11]. These differences in the positive predictive value of PI-RADS 3, 4, and 5 lesions may be partly explained by differences in regional PCa prevalence, mpMRI quality and reporting, and the applied biopsy protocol [10, 11]. Nevertheless, the range of the reported positive predictive value of mpMRI findings in the literature also suggests differences in the individual risk for csPCa, which might be explained by other clinical characteristics [12, 13].

Predicting the individual risk for csPCa before biopsy in men with suspicious mpMRI lesions may help patients and the treating urologist in their shared decision-making regarding the biopsy strategy and in preparing for the biopsy outcome. To date, there are several publicly available PCa risk calculators for calculating individual PCa risk. However, most of these calculators do not consider important clinical features, such as prostate volume and mpMRI result [14–17]. Therefore, this study aimed to calculate a multivariable risk model incorporating several clinical features and PI-RADS for predicting csPCa on prostate biopsy using a large sample of MRI-targeted biopsies.

## Methods

### Patients

For this analysis, data from 701 patients undergoing prostate biopsies between November 2019 and August 2023 at the *Department of Urology of Klinikum rechts der Isar of Technical University of Munich* was assessed. Among these, 24 were excluded due to a prior PCa diagnosis, acute prostatitis, ASAP, adenocarcinoma, or prostate imagining with just PSMA-PET CT, yielding 677 patients for univariable patient characteristic summaries. For multivariable analysis, a further 74 patients were excluded with missing PSA (2 patients), PI-RADS score of the index lesion (1), or prostate volume (71), yielding 603 patients for analysis. Approval for the analysis of anonymized patient data was obtained by the TUM Ethics board.

### mpMRI and biopsy procedure

A mpMRI was performed before all biopsies. All mpMRI scans were interpreted by an experienced radiologist according to the PI-RADS V.2.0 protocol. The Canon’s Aplio I800 ultrasound system with software fusion of MRI and live ultrasound images was used for all biopsies. 95.2% of biopsies were transrectal, and 4.8% were transperineal under local anesthesia. An average of 3 cores per mpMRI lesion were obtained, followed by a systematic 10–12 core biopsy (5–6 from each lobe targeting the peripheral zone and transitional zone of the base, midportion, and apex of the prostate) to obtain biopsy cores from the radiologically non-suspicious areas of the prostate.

### Outcomes

Clinically significant prostate cancer (csPCa) was primarily defined as ISUP ≥ 3 and was considered the primary outcome for analysis, with all analyses repeated for csPCa defined as ISUP ≥ 2.

### Characteristics

The following clinical characteristics were assessed: Age, PSA, prostate volume, PI-RADS score of index lesion, digital rectal examination (DRE), prior negative biopsy, biopsy type (systematic only, targeted only, targeted + systematic), biopsy method (transperineal, transrectal), number of biopsy cores.

### Statistical methods

Univariable differences between csPCa and non-csPCa were assessed using Fisher’s exact test for categorical and Wilcoxon–Mann–Whitney test for continuous variables with p values reported for a test of the null hypothesis of no association. Multivariable logistic regression was used to assess the independent association of multiple risk factors, including the risk factors that minimized the Bayesian Information Criterion (BIC). To increase the fit of the models, PSA density was log-base-2 transformed so that resulting odds ratios were interpreted as the change in odds for doubling their values. Age was divided by 10 to represent 10-year steps. Alternative models treating age and PSA density as categorical variables were considered but obtained higher BIC values, so they dropped from further consideration. The multivariable logistic regression models were fit for patients with all available data. The BIC-selected model was refit to all patients using multiple imputation for the missing risk factors and yielded near identical estimates and significance. All statistical calculations were performed in the R statistical package with significance determined at the two-sided 0.05 level.

## Results

Among the 677 patients, 212 (35.2%) were diagnosed with csPCa (ISUP ≥ 3). These patients were significantly older (71 [64–77] years vs. 64 [57–71] years), had higher PSA (8.3 [5.9–13.6] ng/ml vs. 6.5 [4.8-9.0] ng/ml), had lower prostate volume (40.0 [33.8–54.2] ml vs. 45.0 [35.0–64.8] ml), had higher index PI-RADS score (PI-RADS 4 or 5 vs. 1–3: 94.8% vs. 66.9%), were more likely to have an abnormal DRE (49.5% vs. 21.8%) and were less likely to have a prior negative biopsy (7% vs. 14.5%) (all *p* < 0.05) (Table 1). Results were similar for stratification by csPCA defined as ISUP ≥ 2 (Supplementary Appendix).

**Table 1:** Clinical characteristics of the study sample and differences between non-csPCa and csPCa patients

Characteristic	Total (*n* = 677)		non-csPCa (*n* = 465)		csPCa (*n* = 212)		*P* value
Median age at biopsy (years) (IQR) (*n* = 677; missing: 0)	67 (59–73)		64 (57–71)		71 (64–77)		< 0.001
Median PSA (ng/ml) (IQR) (*n* = 675; missing: 2)	7.1 (5.1–10.4)		6.5 (4.8-9.0)		8.3 (5.9–13.6)		< 0.001
Median prostate volume (ml) (IQR) (*n* = 606; missing: 71)	45 (35–60)		45 (35.0-64.8)		40 (33.8–54.2)		0.01
No. max. PI-RADS Score (%) (*n* = 676; missing: 1)							< 0.001
≤ 2	37 (5.5)		36 (8.1)		1 (0.4)		
3	123 (18.2)		112 (25.1)		11 (4.8)		
4	338 (50.0)		227 (50.8)		111 (48.5)		
5	178 (26.3)		72 (16.1)		106 (46.3)		
No. DRE (%) (*n* = 597; missing: 80)							< 0.001
Suspicious	186 (31.2)		86 (21.8)		100 (49.5)		
Not suspicious	411 (68.8)		309 (78.2)		102 (50.5)		
No. prior biopsy (%) (*n* = 677; missing: 0)							0.004
Yes (negative)	81 (12.0)		65 (14.5)		16 (7.0)		
No	596 (88.0)		383 (85.5)		213 (93.0)		
No. biopsy type (%) (*n* = 677; missing: 0)							0.219
Systematic only	5 (0.7)		5 (1.1)		0 (0.0)		
Targeted only	54 (8.0)		33 (7.4)		21 (9.2)		
Targeted + systematic	618 (91.3)		410 (91.5)		208 (80.8)		
No. biopsy method (%) (*n* = 677; missing: 0)							1.0
Perineal	33 (4.9)		22 (4.9)		11 (4.8)		
Transrectal	644 (95.1)		426 (95.1)		218 (95.2)		
Median number of total cores extracted (Range) (*n* = 677; missing: 0)	15 (2–22)		15 (2–22)		15 (3–20)		0.264

*csPCa* clinically significant prostate cancer, *DRE* digital rectal examination, *IQR* interquartile range, *No.* number, *PSA* prostate-specific antigen, *SD* standard deviation

The csPCa detection rate in patients with a PI-RADS 3, 4, and 5 index lesion was 8.9% (11/123), 32.8% (111/338), and 59.5% (106/178), respectively. The detection rate for csPCA, defined as ISUP ≥ 2, was 31.7% (39/123), 75.4% (255/338), and 92.6% (165/178), respectively.

Based on the 603 patients with all risk factors available, older age (OR: 1.64 for a 10-year increase; CI [1.3-2.0]; *p* < 0.001), higher PSA density (OR: 1.60 for a doubling; CI [1.3–1.9]; *p* < 0.001), higher PI-RADS score of the index lesion (OR: 2.35 for an increase of 1; CI [1.8–3.1]; *p* < 0.001), and a prior negative biopsy (OR: 0.43; CI [0.2–0.8]; *p* = 0.01) were associated with csPCa, while digital rectal exam, biopsy type (transrectal versus perineal), biopsy method (systematic only, targeted only or targeted + systematic), and cores taken were not (all *p* > 0.05). Multiple imputations for missing risk factors to analyze the entire cohort feasibly yielded near-identical results. Odds ratios were similar for multivariable models for csPCA defined as ISUP ≥ 2 (Supplementary Appendix).

**Fig. 1:**
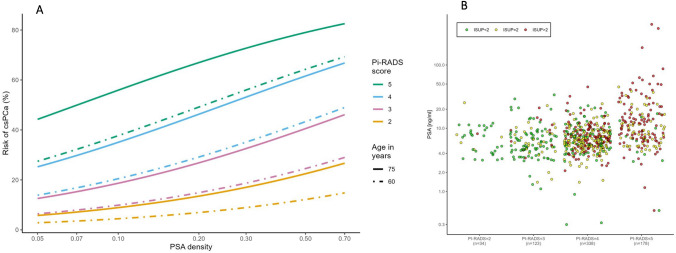
**A** Model-based risk curves for different PI-RADS scores (from top to bottom in decreasing order) of age groups 60 and 75 years over PSA density; no prior negative biopsy was assumed. **B** ISUP scores in relation to PSA versus PI-RADS scores (*n* = 673 patients)

Figure 1A shows the model’s risk curves for an example patient set. Depicted are the risk curves for csPCa of a 60-year-old and a 75-year-old patient stratified by PI-RADS score over PSA density, illustrating that independent of the PI-RADS score, younger patients and patients with a lower PSA density have a lower risk of csPCa. For a 60-year-old patient, the risk curve lies closely to that of a 75-year-old patient with a PI-RADS score decreased by one unit.

The relation between PSA and PI-RADS is shown in Fig. 1B. The variability of PSA and the prevalence of ISUP ≥ 2 increases for higher PI-RADS scores.

## Discussion

mpMRI is regularly used to assess men with elevated PSA to stratify the individual risk of csPCa before performing an invasive biopsy and risking over-detection of clinically non-significant PCa [3, 5, 18, 19]. The high sensitivity of mpMRI allows omitting a biopsy in most men with unsuspicious mpMRI (PI-RADS < 3) [4, 5, 19]. However, the reported positive predictive value of a suspicious mpMRI shows significant variability between different studies [10, 11]. Furthermore, most publicly available prostate cancer risk calculators allow for prior positive cancer diagnoses or do not include prostate volume or mpMRI results [14, 16, 17]. In this analysis, we aimed to evaluate additional clinical risk factors for the detection of csPCa, to further analyze their impact on the predictive value of mpMRI, and to develop a contemporary risk tool for predicting csPCa prior to biopsy, which we plan to make publicly available for further external validation. As expected, our analysis identified older age (OR: 1.64 for a 10-year increase; CI [1.3–2.0]; *p* < 0.001), higher PSA density (OR: 1.60 for a doubling; CI: [1.3–1.9]; *p* < 0.001), higher PI-RADS score of the index lesion (OR: 2.35 for an increase of 1; CI [1.8–3.1]; *p* < 0.001), and a prior negative biopsy (OR: 0.43; CI [0.2–0.8]; *p* = 0.01) as risk factors for csPSA regardless of its definition. In this regard, the PI-RADS score emerged as the most significant predictor for csPCa, confirming previous studies highlighting the diagnostic value of mpMRI and underscoring the high risk for csPCa in patients with a PI-RADS 4 or 5 lesions regardless of other clinical characteristics.

In our sample, among men with a PI-RADS 3, 4, and 5 index lesion, csPCa (defined as ISUP ≥ 3) was detected in 8.9%, 32.8%, and 59.5%, and csPCa (defined as ISUP ≥ 2) in 31.7%, 75.4% and 92.6%, respectively. Regarding PI-RADS 5 lesions, the rate of csPCa detection is similar to previous studies. However, the cancer detection rate in patients with a PI-RADS 3 or 4 index lesion was considerably higher than in most previous studies [11]. In this regard, meta-analyses have shown a significant variability of the positive predictive value (PPV) of PI-RADS 3 and 4 lesions. As an explanation for this variability, differences in regional PCa prevalence, mpMRI quality and inter-radiologist variability in mpMRI interpretation have been previously suggested [10, 20]. Nevertheless, the results of our analysis also suggest inter-patient variability in the predictive value of mpMRI for csPCa. This means that a lesion that has all the characteristics to be considered a PI-RADS 4 lesion may be more or less likely to be a sign of prostate cancer, depending on other clinical factors. First, PSA density was identified as a predictor of csPCa independent of mpMRI results. Our results show that the risk of csPCa increases with increasing PSA density in all PI-RADS subgroups. This leads to a doubled risk of csPCa in patients with a PIRADS 3 or 4 lesion and a PSA density of 0.5 (e.g. PSA 10.0 ng/ml and prostate volume of 20 ml) compared to a patient with a PIRADS 3 or 4 lesion and a PSA density of 0.07 (e.g., PSA 4.0 ng/ml and prostate volume of 60 ml).

Moreover, age was also identified as an independent risk factor for detecting csPCa, influencing the mpMRI-based PCa risk. The calculated risk model shows that for a 60-year-old patient, the risk curve lies closely to that of a 75-year-old patient with a 1-point lower PI-RADS score. Previous research has suggested that mpMRI may be challenging to interpret in younger men, highlighting the value of considering other clinical variables when making further clinical decisions [21]. These findings are especially relevant for clinical decision-making in men with a PI-RADS 3 lesion. These men are often considered at risk for csPCa, although the PPV of a PI-RADS 3 lesion has been reported to be as low as 7%. This current analysis shows that factoring in other clinical characteristics, such as age and PSA density, the risk for csPCa might be considerably higher in some men with a PI-RADS 3 than in others with the same mpMRI findings. Considering these standard clinical values may facilitate decision-making in patients with PI-RADS 3 lesions and support an observational approach without an invasive biopsy in patients with a low risk for csPCa [22]. For example, in younger men with a larger prostate, a lower PSA density, and a PI-RADS 3 lesion, the lesion is likely to be benign. However, in older men with a PSA density greater than 0.5 (e.g., PSA 10.0 ng/ml and a prostate volume of 20 ml) and a PI-RADS 3 lesion, the risk of csPCa may be as high as 30–40%.

Regarding patients with PI-RADS 4 or 5 lesions, the significant risk for csPCa, regardless of other clinical characteristics, makes a further diagnostic evaluation necessary in most cases. Nevertheless, considering the above clinical characteristics, there appears to be a substantial difference in the individual risk for csPCa, even among patients with PI-RADS 4 or 5 lesions. Discussion of individual csPCa risk prior to biopsy may help patients prepare for the biopsy result and influence clinical decision making regarding biopsy strategy. In patients with a high probability of csPCa, a reduced number of extracted biopsy cores or a targeted biopsy-only approach might be feasible [23–25]. Further, in case of a negative biopsy, these factors might help in the decision-making regarding an early follow-up mpMRI and biopsy or a less strict observational follow-up.

Several limitations of this study must be considered. First, given its retrospective design, the results of this study are prone to selection bias. Still, as no case was excluded during the investigation, potential selection bias was minimized. Second, most prostate biopsies included in this analysis were performed transrectally. Therefore, the results of this analysis might not be comparable to a transperineal approach. Although the PCa detection rate has been reported to be slightly higher when comparing the transperineal with the transrectal biopsy approach, a significant procedural bias seems unlikely [26]. Third, our results are based on data from a high-volume prostate cancer center, resulting in a high prevalence of csPCa in our data sample, which is higher compared to most prostate biopsy data samples. When comparing the results of this analysis with the most recent MRI-based risk calculators, similar odds ratios can be obtained when adjusting for comparable risk factors [14, 15]. However, our risk model predicts the highest probability of csPCa. Therefore, our calculated risk models may be less applicable in samples with lower PCa incidence. Finally, our risk model does not consider the experience level of the radiologist and the urologist performing the biopsy. Biopsy experience is a known factor influencing biopsy accuracy, particularly with targeted biopsy. However, recent results have shown that combining targeted and systematic biopsies facilitates the overall csPCa detection rate in less experienced urologists [23]. Furthermore, the widespread implementation of the PI-RADS V.2.0 protocol has helped reduce inter-radiologist variability when assessing mpMRIs [27–29]. Therefore, we believe that our results and the utility of our risk model are broadly applicable.

In conclusion, PI-RADS is the dominant predictor for csPCa on follow-up prostate biopsy. However, PSA density, age, and prior negative biopsy history contribute independent predictive value and must be considered when discussing the individual risk for csPCa following mpMRI. These factors should be considered, especially in men with a PI-RADS 3 lesion, to facilitate decision-making regarding further invasive diagnostic evaluation. In men with a PI-RADS 4 or 5 lesion, predicting the risk for csPCa before biopsy more accurately might help prepare for the biopsy outcome and can be helpful when considering the biopsy strategy. To further refine and externally validate the risk model that underlies this analysis, it will be made publicly available as a risk tool for predicting individual prebiopsy csPCa risk.

## Electronic supplementary material

Below is the link to the electronic supplementary material.

Supplementary Material 1

## Data Availability

Data are available for bona fide researchers who request it from the authors.
