# Supplementary material for: Predicting clinically significant prostate cancer following suspicious mpMRI: analyses from a high-volume center
Source: World J Urol. 2024 May 3;42(1):290. doi: 10.1007/s00345-024-04991-6 (PMC11068682; doi:10.1007/s00345-024-04991-6)
Supplement: Supplementary file 1 — Supplementary Material 1 [file 345_2024_4991_MOESM1_ESM.docx]

**Appendix**

**Appendix Table 1:** Clinical characteristics of the study sample, differences between non-csPCa and csPCa patients (defined as ISUP ≥2).

| **Characteristic** | **Total** | | **ISUP < 2** | | **ISUP ≥ 2** | | **P-value** |
| --- | --- | --- | --- | --- | --- | --- | --- |
| **Median age at biopsy *(years)* (IQR)**  (n = 606; missing: 71) | 67 | (59-73) | 62 | (52-70) | 69 | (61-75) | <0.001 |
| **Median PSA (*ng/ml)* (IQR)**  (n = 675; missing: 2) | 7.1 | (5.1-10.4) | 6.4 | (4.5-8.7) | 7.4 | (5.4-11.3) | <0.001 |
| **Median prostate volume (*ml)*** **(IQR)**  (n = 606; missing: 71) | 45 | (35-60) | 54 | (38-75) | 40 | (30-55) | <0.001 |
| **No. max. PI-RADS Score (%)**  (n = 676; missing: 1) |  |  |  |  |  |  | <0.001 |
| ≤2 | 37 | (5.5) | 31 | (14.7) | 6 | (1.3) |  |
| 3 | 123 | (18.2) | 84 | (39.8) | 39 | (8.4) |  |
| 4 | 338 | (50.0) | 83 | (39.3) | 255 | (54.8) |  |
| 5 | 178 | (26.3) | 13 | (6.2) | 165 | (35.5) |  |
| **No. DRE (%)** (n = 597; missing: 80) |  |  |  |  |  |  | <0.001 |
| Suspicious | 186 | (31.2) | 15 | (8.2) | 171 | (41.3) |  |
| Not suspicious | 411 | (68.8) | 168 | (91.8) | 243 | (58.7) |  |
| **No. prior biopsy (%)** (n = 677; missing: 0) |  |  |  |  |  |  | <0.001 |
| Yes (negative) | 596 | (88.0) | 171 | (80.7) | 425 | (91.4) |  |
| No | 81 | (12.0) | 41 | (19.3) | 40 | (8.6) |  |
| **No. biopsy type (%)** (n = 677; missing: 0) |  |  |  |  |  |  | <0.001 |
| Systematic only | 5 | (0.7) | 5 | (2.4) | 0 | (0) |  |
| Targeted only | 54 | (8.0) | 6 | (2.8) | 48 | (10.3) |  |
| Targeted + systematic | 618 | (91.3) | 201 | (94.8) | 417 | (89.7) |  |
| **No. biopsy method (%)**  (n = 677; missing: 0) |  |  |  |  |  |  | 0.012 |
| Perineal | 33 | (4.9) | 4 | (1.9) | 29 | (6.2) |  |
| Transrectal | 644 | (95.1) | 208 | (98.1) | 436 | (93.8) |  |
| **Median number of total cores extracted (IQR)** (n = 677; missing: 0) | 15 | (15-16) | 15 | (15-16) | 15 | (15-16) | 0.239 |
| **Abbreviations:** csPCa, clinically significant prostate cancer; DRE, digital rectal examination; IQR, inter quartile range; ISUP, International Society of Urological Pathology; Min-Max, minimum – maximum, No. number; PSA, prostate specific antigen; SD, standard deviation | | | | | | | |

**Appendix 2:** Risk factors and their odds ratios (ORs), 95% confidence intervals (CIs), and p values of a logistic regression model predicting the probability of ISUP≥2 (n=603 patients).

| **Risk factor** | **OR** | **95%-CI** | **P-value** |
| --- | --- | --- | --- |
| Age in 10-year steps | 1.58 | [1.2-2.0] | <0.001 |
| Log2 PSA density | 2.49 | [1.9-3.3] | <0.001 |
| PIRADS Score | 4.03 | [2.9-5.6] | <0.001 |
| Prior negative biopsy | 0.33 | [0.2-0.6] | <0.001 |


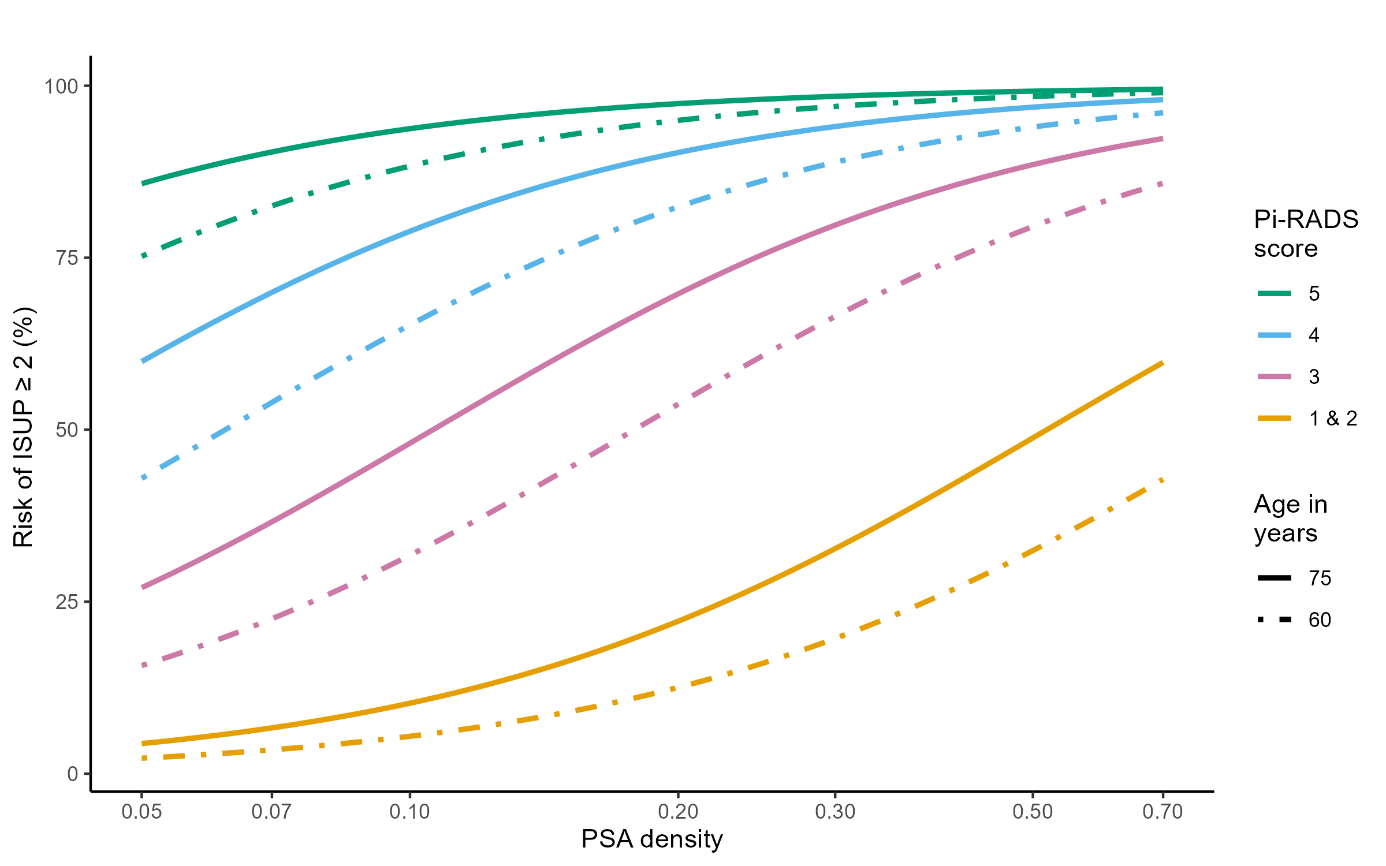


**Appendix Figure 1.** Risk curves ISUP≥2 for different Pi-RADS scores (from top to bottom in decreasing order) of age groups 60 and 75 years over PSA density; no prior negative biopsy was assumed.
